# Supplementary material for: The Handbook of Minerals on a Gluten-Free Diet
Source: Nutrients. 2018 Nov 5;10(11):1683. doi: 10.3390/nu10111683 (PMC6266778; doi:10.3390/nu10111683)
Supplement: Supplementary file 1 [file nutrients-10-01683-s001.pdf]

Table S1. Calcium in gluten–free I – flours, II – mixes for cooking, III – bakery products, IV – cereals and flakes, V – groats and rice, VI – pasta.

	PRODUCT	PRODUCER/INGREDIENTS/OTHER INFORMATION	MG 100 G	MG PORTION	RNI PORTION	REFERENCE
**I**	Pantry	The Gluten–Free Pantry, Inc	500	150	19	[1]
	Amaranth Flour		207 (94–345) [1]	62 (28–104) [1]	8 (4–13) [1]	[2–5]
	Measure for Measure Flour	The King Arthur Flour Company, Inc.	194	58	7	[1]
	Acorn Flour		164	49	6	[5]
	Amaranth Flour Roasted		150	45	6	[3]
	Teff Flour		139 (84–180) [1]	42 (25–54) [1]	5 (3–7) [1]	[3,5–7]
	Chickpea Flour		92	28	3	[5]
	Whole Grain Bread Flour	Sorghum, Teff, Millet, Rice	67	20	3	[1]
	Chestnut Flour		58	17	2	[5]
	Quinoa Flour		34 (18–50) [1]	10 (5–15) [1]	2 (1–2) [1]	[6–8]
	Oat Flour		31 (22–40) [1]	10 (7–12) [1]	2 (1–2) [1]	[5–7]
	Flour	Jyttemel Original	37	11	1	[9]
	Flour, Coarse	Semper	34	10	1	[9]
	Flour		32	10	1	[10]
	Sprouted Yellow Cornmeal	To Your Health Sprouted Flour Co.	30	9	1	[1]
	Flour With 10% Of Oat Flour	Corn, Potato, Oat	26	8	1	[5]
	Mixed Flours		26	8	1	[11]
	Buckwheat Flour		22 (11–40) [1]	6 (3–12) [1]	1 (0–2) [1]	[3,5–7]
	Flour, Mix	Rice, Soya, Corn, Tapioca	20	6	1	[12]
	Millet Flour		14 (13–14) [1]	4 (4) [1]	1 (0–1) [1]	[3,5]
	Flour	Schär	12	4	0	[9]
	Corn Starch		11	3	0	[3]
	Sorghum Flour		10	3	0	[6,7]
	Brown Rice Flour		10	3	0	[13]
	Rice Flour		6 (0–10) [1]	2 (0–3) [1]	0 (0) [1]	[3,5–7,13]

	Rice Starch		5	2	0	[3]
	Corn Flour		3 (0–7) [1]	1 (0–2) [1]	0 (0) [1]	[3,5–7,13]
	No Data For 20 Flours					
**II**	Cornbread Mix	The King Arthur Flour Company, Inc.	513	154	19	[1]
	Baking Mix White	Glutafin	400	120	15	[14]
	Baking Mix Brown	Glutafin	400	120	15	[14]
	All Purpose Baking Mix	New Hope Mills Manufacturing, Inc.	250	75	9	[1]
	Premium Biscuit Mix	Duinkerken Foods Inc.	250	75	9	[1]
	Southern Glory, Biscuits Baking Mix	1–2–3 Gluten Free, Inc.	194	58	7	[1]
	Pancake Mix	Bob's Red Mill Natural Foods, Inc.	190	57	7	[1]
	Pancake Mix	Rice, Tapioca, Corn	162	49	6	[1]
	Muffin Mix	New Hope Mills Manufacturing, Inc.	158	47	6	[1]
	Bisquick Baking Mix Pancake & Waffle Mix	General Mills Sales Inc.	158	47	6	[1]
	Vanilla Cupcake Mix	New Hope Mills Manufacturing, Inc.	121	36	5	[1]
	Pancake + Baking Mix	Schnuck Markets, Inc.	100	30	4	[1]
	Bake Mix (Pizza)		89	27	3	[10]
	Betty Crocker Yellow Cake Mix	General Mills Sales Inc.	85	26	3	[1]
	Fudge Brownie Mi	Arrowhead Mills	80	24	3	[1]
	Cookie Mix, Chocolate Chip	Aldi–Benner Company, LiveGFree	74	22	3	[1]
	Blueberry Muffin Mix	Continental Mills, Inc.	61	18	2	[1]
	Favorite Sandwich Bread Mix	The Gluten–Free Pantry, Inc. Glutino	60	18	2	[1]
	Fluffy Pancake Mix	The Gluten–Free Pantry, Inc. Glutino	57	17	2	[1]
	Bread Mix	Bob's Red Mill Natural Foods, Inc.	57	17	2	[1]
	Bake Mix White (Cake)		55	16	2	[10]
	Wheat–Free Baking Mix, Classic Chocolate Cake	Schnuck Markets, Inc.	57	17	2	[1]
	Cake Base, Powder	Toro	53	16	2	[9]
	Cake Mix	Cup4cup, Llc	50	15	2	[1]

	Yellow Cake Mix	Cup4cup, Llc	50	15	2	[1]
	Yellow Cake Mix	Aldi–Benner Company LiveGFree	47	14	2	[1]
	Flour for Bread		45	14	2	[11]
	Chocolate Cake Mix	The King Arthur Flour Company, Inc.	44	13	2	[1]
	Muffin Mix	Cup4cup, Llc	41	12	2	[1]
	Oat Mix For Bread	Oat (61%), Potato	40	12	2	[5]
	Cookie Mix	Bob's Red Mill Natural Foods, Inc.	38	11	1	[1]
	Brownie Mix with Fudge Frosting	Tapioca, Potato	36	11	1	[1]
	Multigrain Mix For Bread	Corn, Rice, Linseed Flour (6%), Sunflower Seeds (6%)	33	10	1	[5]
	Breadcrumbs		32	10	1	[10]
	Oat Cookies Mix	Oat (45%), Rice, Corn	29	9	1	[5]
	Oat Mix for Chocolate Muffins	Oat (20%), Potato, Rice	25	8	1	[5]
	Lemon Pound Cake Mix	Brandless	25	8	1	[1]
	Stuffing Mix	Marie Antoinette's Gluten Free Bake Shoppe	20	6	1	[1]
	Muffin Mix	Corn, Buckwheat	20	6	1	[5]
	Bake Mix Brown (Cake)		18	5	1	[10]
	Bread Mix	Corn, GF Wheat	17	5	1	[5]
	Flour Mix For Bread		17	5	1	[15]
	Cake Flour Mix		16	5	1	[15]
	Breadcrumbs		15	5	1	[11]
	Cake Mix, Powder	Toro	10	3	0	[9]
	Flour for Cake		7	2	0	[11]
	Baking Mix White	Schar	4	1	0	[14]
	Flour Mix		3	1	0	[15]
	No Data For 30 Products					
**III**	Crisp Bread, Prepared from Powder	Toro	282	282	35	[9]
	Bread White	Glutafin	236	236	30	[14]
	Bread Brown	Glutafin	231	231	29	[14]

Original Sandwich Bread	Rudi's Bakery	180	180	23	[1]
Classic White Rolls	Dr. Schar Usa, Inc.	180	180	23	[1]
Omega Flax Bread	Silver Hills	171	171	21	[1]
Brown Bread	Rice, Corn, Potato	166	166	21	[5]
Rice Bread With Milk	Rice Flour, Potato Starch, Corn Starch, Milk Powder	147	147	18	[16]
Buckwheat Bread With Milk	Buckwheat Flour, Potato Starch, Corn Starch, Milk Powder	142	142	18	[16]
Crispbread		142	142	18	[15]
Rolls	White, Made with Rice Flour, Rice Starch, And Corn Starch	140	140	18	[1]
Bread White	Made with Rice Flour, Corn Starch, and/Or Tapioca	138	138	17	[1]
Rice Bread with Milk and Seeds	Rice Flour, Potato Starch, Corn Starch, Milk Powder, Seeds (Poppy, Amaranth, Flax, Sunflower, Pumpkin, Hazelnuts)	136	136	17	[16]
Buckwheat Bread with Milk and Seeds	Buckhweta Flour, Potato Starch, Corn Starch, Milk Powder, Seeds (Poppy, Amaranth, Flax, Sunflower, Pumpkin, Hazelnuts)	136	136	17	[16]
Sandwich Bread, Whit	Gluten Free Nation	135	135	17	[1]
Seed Crispbread	Water, Buckwheat Flour, Sunflower Seed, Pumpkin Seed, Sesame Seed, Rapeseed Oil, Linseed, Salt	134	134	17	[17]
Swiss Roll,	Egg Without Shell, Sugar, Corn Flour, Potato Starch, Baking Powder, Mixed Berry Jam	132	132	17	[17]
Lye Pretzel		124	124	16	[10]
Whole Grain Dinner Rolls	Udi's Gluten Free	118	118	15	[1]
Rolls, Whole Grain,	Tapioca Starch, Brown Rice Flour	118	118	15	[1]
Seeds & Grains Bread	Glencourt Inc.	105	105	13	[1]
Seeded Whole Grain Dinner Rolls	Pinnacle Foods Group Llc, Udi's Gluten Free	100	100	13	[1]
Wholegrain Bread		96	96	12	[10]
Sprouted for Life, Almond Bread	Food for Life Baking Co Inc	93	93	12	[1]
Flax Bread	Food for Life Baking Co Inc	93	93	12	[1]
3 Seed Bread	Food for Life Baking Co Inc	93	93	1	[1]
Baguette Gourmet Garlic Bread & Subs	Dr. Schar Usa, Inc.	91	91	11	[1]

Classic French Dinner Rolls	Udi's Gluten Free	82	82	10	[1]
Rolls	Brown Rice Flour, Tapioca Starch, Potato Starch	82	82	10	[1]
Sesame Bagels	Katz Gluten Free	72	72	9	[18]
Sprouted Seeds Bread	Grindstone Bakery	70	70	9	[1]
Ancient Grains Bread	Coborn's Gluten Free Bakery	67	67	8	[18]
Bread with Oilseeds		67	67	8	[15]
White Bread with Carob Bean Gum		62	62	8	[15]
Rustic Multigrain Dinner Roll Mix, Seeds & Grains	Bella Gluten–Free Llc	61	61	8	[1]
Bread, Cinnamon–Raisin	The Essential Baking Company, Inc.	61	61	8	[1]
Bread, Whole Grain, Made with Tapioca Starch and Brown Rice Flour		60	60	8	[1]
Soft & Hearty Whole Grain Bread	Pinnacle Foods Group Llc, Udi's Gluten Free	60	60	8	[1]
Bread, With Added Grains, Commercial		60	60	8	[12]
Bread	Tapioca Starch, Brown Rice Flour	59	59	7	[1]
Raisin Bread		59	59	7	[10]
Classic French Dinner Rolls	Pinnacle Foods Group Llc, Udi's Gluten Free	57	57	7	[1]
White Bread	The Kroger Co.	56	56	7	[1]
7 Grain Bread	The Kroger Co.	56	56	7	[1]
Bread, Homemade from Basic Ingredients		55	55	7	[12]
Crisp Bread		53 (6–143) [1]	53 (6–143) [1]	7 (1–18) [1]	[10,11,15]
Bread	GF Wheat, Millet Flour; Corn Starch	52	52	7	[19]
Piadina		52	52	7	[11]
White Sandwich Bread	Pinnacle Foods Group Llc, Udi's Gluten Free	51	51	6	[1]
Bread, Commercial		50	50	6	[12]
Soft & Hearty Whole Grain Bread	Udi's Gluten Free	48	48	6	[18]
Wholesome Bread	Katz Gluten Free	46	46	6	[18]
Bagels, Poppy Seed	Katz Gluten Free	43	43	5	[18]
Wheat–Free, Classic White Bread	Andrea's Gluten Free	41	41	5	[1]

Rolls	Brown Rice Flour, Tapioca Starch, Sorghum Flour	41	41	5	[1]
Soft & Sweet Cinnamon & Raisin Bread	Pinnacle Foods Group Llc, Udi's Gluten Free	41	41	5	[1]
Bread, Flax, Vegan	The Gluten Free Bakery	40	40	5	[18]
Millet Bread		35	35	4	[15]
Toast		34	34	4	[10]
Shortbread		33	33	4	[10]
Crispy & Delicious French Baguettes	Pinnacle Foods Group Llc Udi's Gluten Free	33	33	4	[1]
Artisan Baker White Bread	Dr. Schar Usa, Inc.	32	32	4	[1]
Scone		31	31	4	[10]
Raisin Bread		29	29	4	[15]
Bread	Semper	28	28	4	[9]
Corn Dogs	Foster Poultry Farms	27	27	3	[1]
Oft & Chewy Bagels, Cinnamon Raisin	Pinnacle Foods Group Llc, Udi's Gluten Free	25	25	3	[1]
Soft Dinner Roll	Rudi's Bakery	24	24	3	[1]
Bread, White, Made with Potato Extract, Rice Starch, And Rice Flour		24	24	3	[1]
Bread Crumbs		20	20	3	[15]
White Bread		20	20	3	[15]
Bread	Corn, GF Wheat	19	19	2	[5]
Buckwheat Bread	Buckwheat Flour, Potato Starch, Corn Starch	19	19	2	[16]
Bakehouse, Hamburger Buns	Harry's Farmers Market, Ltd., Whole Foods Market	18	18	2	[1]
Bun		18	18	2	[10]
Ciabatta		17	17	2	[10]
Soft & Delicious White Sandwich Bread	Pinnacle Foods Group Llc, Udi's Gluten Free	17	17	2	[1]
Chestnut Bread		16	16	2	[15]
Rice Bread	Rice Flour, Potato Starch, Corn Starch	15	15	2	[16]
Soft & Hearty Whole Grain Bread	Pinnacle Foods Group Llc, Udi's Gluten Free	15	15	2	[1]
Bagels, Plain	Katz Gluten Free	14	14	2	[18]

	Wholemeal Bread		14	14	2	[11]
	Baquette		14	14	2	[10]
	Melba Toast		13	13	2	[11]
	Rustic Bread		11	11	1	[10]
	Cracklebred	Breton Enterprises Inc	11	11	1	[1]
	Multigrain Bread	Corn, GF Wheat with Linseed, Sunflower, Pumpkin Seeds	11	11	1	[5]
	Brioche Bread		10	10	1	[11]
	Bread Prepared With Olives		10	10	1	[11]
	Bread Prepared With Oil		9	9	1	[11]
	Bread With Grains	Corn, GF Wheat with Linseed and Sunflower Seeds	9	9	1	[5]
	White Bread II		8	8	1	[11]
	Bread		8	8	1	[20]
	Multigrain Bread	Corn, GF Wheat with Linseed, Sunflower, Soya, And Pumpkin Seeds	7	7	1	[5]
	Crisp Bread	Fette Croccanti	7	7	1	[14]
	White Bread I		6	6	1	[11]
	Ciabatta Bread		6	6	1	[11]
	Sliced White Bread (Sandwich)		5	5	1	[11]
	No Data For 21 Products					
**IV**	Breakfast Cereals, Nesquik	Nestlé Belgilux S.A., Fortified	561	168	21	[21]
	Cerelac	Nestlé Belgilux S.A., Fortified	400	120	15	[21]
	Amaranth And Quinoa Flakes		276	83	10	[3]
	Amaranth Flakes		248	74	9	[3]
	Musli With Amaranth	Corn, Rice, Amaranth, Millet, Raisins, Raspberry	237	71	9	[5]
	All–Purpose Flour	The King Arthur Flour Company, Inc.	200	60	8	[1]
	Kellogg Special K Cereal	Kellogg Company Us, Fortified with Fe	163	49	6	[1]
	Granola	Oat, Honey, Flax, Sesame, Pumpkin, Sunflower	143	43	5	[1]
	Oats	Glutenfreeda Foods, Inc.	100	30	4	[1]
	Muesli	Muesli Fusion, Inc.	98	29	4	[1]

Freedom Muesli	Surf City Cereal	95	29	4	[1]
Musli		95	29	4	[11]
Amaranth Flakes		92	28	3	[5]
Granola (including Apple Almond Honey Granola)	Glutenfreeda Foods, Inc.	71	21	3	[1]
Granola	Georgia Peach Products, Inc., Mirracole Morsels	71	21	3	[1]
Cranberry Nut Crisp Granola	Delhaize America, Inc.	71	21	3	[1]
Granola	Garden Of Light Inc.	70	21	3	[1]
Granola (including Original Granola, Au Natural Granola, Cherry Walnut Granola Clusters, Blueberry Cashew Granola Clusters)	Pinnacle Foods Group Llc, Udi's Gluten Free	67	20	3	[1]
Wheat Free, Instant Oatmeal, Strawberries	Glutenfreeda Foods, Inc.	67	20	3	[18]
Oatmeal	Bob's Red Mill Natural Foods, Inc.	64	19	2	[1]
Oatmeal	Topco Associates, Inc.	64	19	2	[1]
Rolled Oats	Glutenfreeda Foods, Inc.	64	19	2	[18]
Amaranth Musli With Fruits	Oat, Amaranth (20%), Fruits (20%), Hazelnut, Sesame, Buckwheat	62	19	2	[5]
Bread Mix	Hodgson Mill	61	18	2	[1]
Granola, Apple Almond Honey	Glutenfreeda	57	17	2	[18]
Muesli, Commercial		57	17	2	[12]
Superfood Oatmeal	Nature's Path Organic	56	17	2	[1]
Organic Superfood Oatmeal	Oat, Coconut, Buckwheat, Chia	53	16	2	[1]
Oatmeal, Cinnamon Pumpkin Seed	Nature's Path Foods Inc.	53	16	2	[1]
Millet Musli		50	15	2	
Oat Bran	Bob's Red Mill Natural Foods, Inc.	50	15	2	[1]
Homestyle Hot Oatmea	Nature's Path Foods Inc.	50	15	2	[1]
Hot Oatmeal, Brown Sugar Maple with Ancient Grains	Oat, Amarantg, Quinoa Sorghum	50	15	2	[1]
Instant Oatmeal	The Quaker Oats Company	47	14	2	[1]
Old Fashioned Oats Oatmeal	Topco Associates, Inc	46	14	2	[1]

	Granola (Nuts)		45	13	2	[10]
	Oat Musli With Fruits	Oat, Fruits, Dates, Raisins, Sunflower Seeds	44	13	2	[5]
	Mixed Berry Instant Oatmeal	Topco Associates, Inc.	43	13	2	[1]
	Instant Oatmeal	Glutenfreeda	43	13	2	[18]
	Oatmeal (including Strawberries & Brown Sugar W/ Flax Oatmeal, Blueberries & Cinnamon W/ Flax Oatmeal)	Glutenfreeda	40	12	2	[18]
	Oats (including Maple Raisin with Flax, Steel Cut Oats)	Glutenfreeda	40	12	2	[18]
	Oat Flakes		34	10	1	[5]
	Instant Oatmeal, Natural	Glutenfreeda	32	10	1	[18]
	4–Grain Flakes	Oat Flakes, Corn Flakes, Rice Flour, Miller Flour	28	8	1	[17]
	Wild Rice Flakes	From Greece	27	8	1	[22]
	Wild Rice Flakes	From Cambodia	26	8	1	[22]
	Wild Rice Flakes	From Hungary	22	7	1	[22]
	Granola (Chocolate)		18	5	1	[10]
	Wild Rice Flakes	From Canada	17	5	1	[22]
	Buckwheat Flakes		15	4	1	[5]
	Sorghum Flakes	Variety: Liberty	13	4	0	[23]
	Millet Flakes		12	4	0	[5]
	Sorghum Flakes	Variety: Is8237c and Alpha	11 (11) [1]	3 (3) [1]	0 (0) [1]	[23]
	Rice Flakes		9	3	0	[5]
	Cornflakes		9 (8–9) [1]	3 (2–3) [1]	0 (0) [1]	[3,10]
	No Data For 8 Products					
**V**	Culinary Ancient Grains	Quinoa Corporation	169	85	11	[3]
	Ancient Quinoa Harvest Quinoa	Quinoa Corporation	159	80	10	[3]
	Steel Cut Oats		120	60	8	[5]
	Happy Quick Oats	Garden Of Light Inc.	74	37	5	[1]
	Whole Grain Steel Cut Oats	Bob's Red Mill Natural Foods, Inc.	61	31	4	[3]

	Old Fashioned Oats	Country Choice Organic	44	22	3	[1]
	White Rice		32	16	2	[3]
	Brown Rice		30	15	2	[1]
	Enriched White Rice		29	15	2	[1]
	Amaranth Popping		28	14	2	[24]
	Rice Cakes With Quinoa	Rice, Quinoa (5%)	28	14	2	[3]
	Couscous		26	13	2	[3]
	Corn Couscous		25	13	2	[3]
	Brown Rice Snack	Brown Rice	23	12	1	[24]
	Brown Rice & Chestnut Snack	Brown Rice, Chestnut Flour (20%)	22	11	1	[5]
	Brown Rice & Chestnut Snack	Brown Rice, Chestnut Flour (40%)	10	5	1	[3]
	Brown Rice & Chestnut Snack	Brown Rice, Chestnut Flour (60%)	10	5	1	[3]
	Rough Rice		8	4	1	[25]
	Milled Rice		8	4	1	[25]
	Tapioca		7	4	0	[25]
	Amaranth Groats		6	3	0	[25]
	Cous–Cous		5 (4–6) [1]	3 (2–3) [1]	0 (0) [1]	[10,11]
	No Data For 7 Products					
**VI**	Pasta	The Hain Celestial Group, Inc. Deboles	70	35	4	[1]
	Rice Lasagna	The Hain Celestial Group, Inc.	56	28	4	[1]
	Pasta With Teff	Corn, Teff (31%)	56	28	3	[5]
	Soya Pasta		51	26	3	[3]
	Vermicelli		45	23	3	[10]
	Tagliatelli		42	21	3	[10]
	Pasta	Millet–Pomace	42	21	3	[26]
	Egg Pasta		38	19	2	[11]
	Organic Elbows	Euro–Usa Trading Co., Inc.	35	18	2	[1]
	Linguini	Euro–Usa Trading Co., Inc. , Bionaturae	35	18	2	[1]
	Lasagne Sheets		31	16	3	[10]

Pasta	Extruded Rice (75%), Amaranth (25%)	30	15	2	[27]
Pasta	Rice (75%), Amaranth (25%)	30	15	2	[27]
Pasta	Extruded Rice (75%), Extruded Amaranth (25%)	29	14	2	[27]
Buckwheat Pasta		26	13	2	[3]
Fresh Fusilli	Rp's Fresh Pasta	24	12	2	[1]
Penne		23	12	1	[10]
Fresh Linguini	Rp's Fresh Pasta	20	10	1	[1]
Brown Rice Pasta		18	9	1	[5]
Spaghetti		18	9	1	[10]
Rice Pasta		17	9	1	[3]
Pasta,		16 (15–16) [1]	9 (8–10) [1]	1 (0–1) [1]	[12,15]
Fusilli		15	8	1	[10]
Pasta, Made of Rice and Vegetables	Orgran Tricolore	14	7	1	[9]
Pasta, Spaghetti	Semper	14	7	1	[9]
Pasta With Buckwheat Flour	Corn, Rice, Buckwheat (15%	14	7	1	[5]
Wholemeal Pasta		12	6	1	[11]
Pasta	Brown Rice (87%), Corn (13%)	8	4	1	[13]
Pasta D'oro Linguine	Corn, Potato, Lupin	7	4	0	[1]
Pasta Made with Different Flours		7	4	0	[11]
Pasta for Broth		7	4	0	[11]
Corn Pasta		5 (4–6) [1]	3 (2–3) [1]	0 (0) [1]	[1,3,11]
Rice Pasta		6	3	0	[11]
Pasta	Schar	6	3	0	[14]
Pasta	Brown Rice (60%), Corn (40%)	6	3	0	[13]
Pasta, Made of Corn And Rice	Barilla	5	3	0	[9]
Pasta	Corn, Rice	4	2	0	[5]
Pasta	Rice	4	2	0	[27]
Pasta	Extruded Rice	3	2	0	[27]
No Data For 27 Products					

[1] The average, minimum and maximum content/RNI of mineral in the same product from different references.

Table S2. Iron in I – flours, II – mixes for cooking, III – bakery products, IV – cereals and flakes, V – groats and rice, VI – pasta.

	PRODUCT	PRODUCER/INGREDIENTS/OTHER INFORMATION	MG 100 G	MG PORTION	RNI PORTION	REFERENCE
I	Acorn Flour		18.6	5.6	40	[5]
	Teff Flour		8.6 (7.6–9.8) [1]	2.6 (2.3–2.9) [1]	18 (16–21) [1]	[3,5–7]
	Quinoa Flour		8.3 (5.4–11.2) [1]	2.5 (1.6–3.4) [1]	18 (11–24) [1]	[6–8]
	Pantry	The Pantry, Inc	9.0	2.7	19	[1]
	Flour, Coarse	Semper	8.5	2.6	18	[9]
	Amaranth Flour		7.9 (6.0–8.4) [1]	2.4 (1.8–2.5) [1]	17 (13–18) [1]	[2]
	Amaranth Flour Roasted		7.5	2.3	16	[3]
	Measure for Measure Flour	The King Arthur Flour Company, Inc.	5.8	1.7	12	[1]
	Chickpea Flour		4.6 (4.3–4.9) [1]	1.4 (1.3–1.5) [1]	10 (9–11) [1]	[3,5]
	Buckwheat Flour		4.3 (1.8–8.3) [1]	1.3 (0.5–2.5) [1]	9 (4–18) [1]	[3,5–7]
	Oat Flour		3.5 (1.6–5.5] [1]	1.0 (0.5–1.6) [1]	8 (4–12) [1]	[3,5–7]
	Millet Flour		3.4 (3.1–3.7) [1]	1.0 (0.9–1.1) [1]	8 (7–9) [1]	[3,5]
	Wholesome Flour	Cup4cup, Llc	3.1	0.9	7	[1]
	Sorghum Flour		2.7	0.8	6	[6,7]
	Flour	Jyttemel Original	2.5	0.8	5	[9]
	Bread Flour		2.4	0.7	5	[1]
	Veganic Sprouted Corn Flour	Veganic	2.4	0.7	5	[1]
	Whole Grain Bread Flour		2.4	0.7	5	[1]
	Chestnut Flour		1.8 (1.2–2.4) [1]	0.6 (0.4–0.7) [1]	4 (3–5) [1]	[3,5]
	Flour		1.4	0.4	3	[10]
	Flour, Mix	Rice, Soy, Corn, Tapioca	1.3	0.4	3	[12]
	Banana Flour	Wedo Entrepreneurs, Llc	1.2	0.4	3	[1]

	Pastry Flour	Sorghum, Rice, Teff, Millet, Corn	1.2	0.4	3	[1]
	Flour With 10% Of Oat Flour	Corn, Potato, Oat	1.2	0.3	2	[5]
	Rice Flour		1.1 (0.4–2.0) [1]	0.3 (0.1–0.6) [1]	2 (1–4) [1]	[3,5–7,24]
	Brown Rice Flour		1.0	0.3	2	[13]
	All–Purpose Flour		0.9	0.3	2	[1]
	Corn Flour		0.6 (0.1–0.9)[1]	0.2 (0.0–0.3)[1]	1 (0–2)[1]	[5–7,13]
	Flour	Schär	0.6	0.2	1	[9]
	Rice Starch		0.4	0.1	1	[3]
	Corn Starch		0.2	0.1	0	[3]
	Mixed Flours		0.0	0.0	0	[11]
	No Data For 11 Products					
**II**	Cornbread Mix	The King Arthur Flour Company, Inc.	6.9	2.1	15	[1]
	Chocolate Cake Mix	The King Arthur Flour Company, Inc.	6.0	1.8	13	[1]
	Brownie Mix	Aldi–Benner Company	6.0	1.8	13	[1]
	Cake Mix	Cup4cup, Llc	5.5	1.7	12	[1]
	Cake Mix	Bob's Red Mill Natural Foods, Inc.	5.1	1.5	11	[1]
	Brownie Mix		5.1	1.5	11	[1]
	Brownie Mix, Double Chocolate	The Pantry, Inc. Glutino	4.9	1.5	10	[1]
	Brownie Mix	Bob's Red Mill Natural Foods, Inc.	4.9	1.5	10	[1]
	Southern Glory, Biscuits Baking Mix	1–2–3 , Inc.	4.7	1.4	10	[1]
	Premium Biscuit Mix	Duinkerken Foods Inc.	4.5	1.4	10	[1]
	Bread Mix	Bob's Red Mill Natural Foods, Inc.	4.1	1.2	9	[1]
	GF Wheat–Free Baking Mix, Classic Chocolate Cake	Schnuck Markets, Inc.	4.1	1.2	9	[1]
	Baking Mix White	Glutafin	4.0	1.2	9	[14]
	Baking Mix Brown	Glutafin	4.0	1.2	9	[14]

Betty Crocker Chocolate Brownie Mix	General Mills Sales Inc.	3.9	1.2	8	[1]
Immaculate Baking Company Double Chocolate Cookie Mix	General Mills Sales Inc.	3.6	1.1	8	[1]
Cookie Mix	Bob's Red Mill Natural Foods, Inc.	3.5	1.0	7	[1]
Betty Crocker Devil's Food Cake Mix	General Mills Sales Inc.	3.4	1.0	7	[1]
Chewy Chocolate Brownie Mix	Bella Llc	3.3	1.0	7	[1]
Organic Bake Mix	Mr. Beverages Old Time Cocktail Mixes	3.1	0.9	7	[1]
Oat Mix for Chocolate Muffins	Oat (20%), Potato, Rice	3.0	0.9	6	[5]
Oat Cookies Mix	Oat (45%), Rice, Corn	3.0	0.9	6	[5]
Fudge Brownie Mi	Arrowhead Mills	2.9	0.9	6	[1]
Oat Mix For Bread	Oat (61%), Potato	2.8	0.8	6	[5]
Pancake + Baking Mix	Schnuck Markets, Inc.	2.7	0.8	6	[1]
Bread Mix	Bob's Red Mill Natural Foods, Inc.	2.6	0.8	6	[1]
Divinely Decadent, Silky & Rich Brownies Baking Mix	1–2–3 , Inc.	2.5	0.7	5	[1]
Breadcrumbs		2.3	0.7	5	[10]
Fluffy Pancake Mix	The Pantry, Inc. Glutino	2.1	0.6	4	[1]
Cookie Mix	The King Arthur Flour Company, Inc.	1.9	0.6	4	[1]
Muffin Mix	Cup4cup, Llc	1.8	0.6	4	[1]
All Purpose Baking Mix	New Hope Mills Manufacturing, Inc.	1.8	0.5	4	[1]
Bake Mix (Pizza)		1.5	0.4	4	[10]
Multigrain Mix For Bread	Corn, Rice, Linseed Flour (6%), Sunflower Seeds (6%)	1.4	0.4	3	[5]
Cookie Mix, Chocolate Chip	Aldi–Benner Company	1.3	0.4	3	[1]
Betty Crocker Chocolate Chip Cookie Mix	General Mills Sales Inc.	1.3	0.4	3	[1]

Cake Base, Powder	Toro	1.3	0.4	3	[9]
Cornbread Mix	Cup4cup, Llc	1.3	0.4	3	[1]
Sugar Cookie Mix	The King Arthur Flour Company, Inc.	1.3	0.4	3	[1]
Bake Mix Brown (Cake)		1.2	0.4	3	[10]
Bake Mix White (Cake)		1.2	0.4	3	[10]
Vanilla Cupcake Mix	New Hope Mills Manufacturing, Inc.	1.1	0.3	2	[1]
Blueberry Muffin Mix	Continental Mills, Inc.	1.1	0.3	2	[1]
Bisquick Baking Mix Pancake & Waffle Mix	General Mills Sales Inc.	1.1	0.3	2	[1]
Breadcrumbs		1.0	0.3	2	[11]
Cornbread Mix	Bob's Red Mill Natural Foods, Inc.	1.0	0.3	2	[1]
Baking Mix, Chocolate Cake	The Kroger Co.	1.0	0.3	2	[1]
Muffin Mix	Corn, Buckwheat	1.0	0.3	2	[5]
Pancake Mi		1.0	0.3	2	[1]
Muffin Mix	New Hope Mills Manufacturing, Inc.	1.0	0.3	2	[1]
Muffin Mix	Namaste Foods	1.0	0.3	2	[1]
Biscuits, Piecrust & More Baking Mix	Namaste Foods	1.0	0.3	2	[1]
Flour For Cakes		0.9	0.3	2	[28]
All Purpose Baking Mix	The Kroger Co.	0.9	0.3	2	[1]
Flour Mix For Bread		0.9	0.3	2	[15]
Pancake Mix	Giant Eagle, Inc.	0.9	0.3	2	[1]
Pancake Mix	Bob's Red Mill Natural Foods, Inc.	0.9	0.3	2	[1]
Cake Flour Mix		0.8	0.2	3	[15]
Yellow Cake Mix	Aldi–Benner Company	0.8	0.3	2	[1]
Spice Cake Mix	Namaste	0.8	0.2	2	[1]
Yellow Cake Mix	Cup4cup, Llc	0.8	0.2	2	[1]
Stuffing Mix	Marie Antoinette's Bake Shoppe	0.7	0.2	2	[1]

	Brownie Mix with Fudge Frosting		0.7	0.2	1	[1]
	Cake Mix, Powder, Toro	Toro	0.4	0.1	1	[9]
	Bread Mix	Corn, GF Wheat	0.3	0.1	1	[5]
	Baking Mix White	Schar	0.1	0.0	0.0	[14]
	Flour Mix		0.0	0	0	[15]
	Flour for Cake		0.0	0	0	[11]
	Flour for Bread		0.0	0	0	[11]
	No Data for 10 Products					
**III**	Crisp Bread, Prepared from Powder	Toro	7.0	7.0	50	[9]
	Brown Bread	Rice, Corn, Potato	6.9	6.9	49	[5]
	Shortbread		4.8	4.8	34	[28]
	Local Bread P.		4.2	4.2	30	[28]
	Seed Crispbread	Water, Buckwheat Flour, Sunflower Seed, Pumpkin Seed, Sesame Seed, Rapeseed Oil, Linseed, Salt	4.1	4.1	29	[17]
	Wholesome Bread,	Katz	4.0	4.0	29	[18]
	Local Bread B.		4.0	4.0	29	[28]
	Sprouted Seeds Bread	Grindstone Bakery	3.8	3.8	27	[1]
	Original Sandwich Bread	Rudi's Bakery	3.6	3.6	26	[1]
	Classic White Rolls	Dr. Schar Usa, Inc.	3.6	3.6	26	[1]
	Baguette Gourmet Garlic Bread & Subs	Dr. Schar Usa, Inc.	3.3	3.3	23	[1]
	Omega Flax Bread	Silver Hills	3.1	3.1	22	[1]
	Bread, Deli Slice Classic White	The Essential Baking Company, Inc.	3.1	3.1	22	[1]
	Ancient Grains Bread	Coborn's Bakery	2.9	2.9	21	[18]
	Rolls, White, Made With Rice Flour, Rice Starch, And Corn Starch		2.8	2.8	20	[1]

	Bread, White, Made With Rice Flour, Corn Starch, And/Or Tapioca		2.6	2.6	19	[1]
	Bread White	Glutafin	2.6	2.6	19	[14]
	Flax Bread	Food for Life Baking Co Inc	2.5	2.5	18	[1]
	Bread Brown	Glutafin	2.5	2.5	18	[14]
	Millet Bread		2.4	2.4	17	[15]
	Wholegrain Bread		2.4	2.4	17	[10]
	Rustic Multigrain Dinner Roll Mix, Seeds & Grains	Bella Llc	2.2	2.2	16	[1]
	Bread with Grains	Corn, GF Wheat with Linseed, And Sunflower Seeds	2.2	2.2	16	[5]
	Bread Crumbs		2.1	2.1	15	[15]
	Buckwheat Bread with Milk And Seeds	Buckwheat Flour, Potato Starch, Corn Starch, Milk Powder, Seeds (Poppy, Amaranth, Flax, Sunflower, Pumpkin, Hazelnuts)	2.1	2.1	15	[16]
	Multigrain Bread	Corn, GF Wheat With Linseed (2.7%), Sunflower (2.7%), And Pumpkin Seeds (2.7%)	2.1	2.1	15	[5]
	Sesame Bagels	Katz	1.9	1.9	14	[18]
	Seeds & Grains Bread	Glencourt Inc.	1.9	1.9	14	[1]
	Bread with Oilseeds		1.9	1.9	14	[15]
	Multigrain Bread	Corn, GF Wheat with Linseed, Sunflower, Soya, And Pumpkin Seeds	1.8	1.8	13	[5]
	Seeded Whole Grain Dinner Rolls	Pinnacle Foods Group Llc	1.8	1.8	13	[1]
	Buckwheat Bread with Milk	Buckwheat Flour, Potato Starch, Corn Starch, Milk Powder	1.7	1.7	12	[16]
	Sprouted for Life, Almond Bread	Food for Life Baking Co Inc	1.7	1.7	12	[1]
	Original 3 Seed Bread	Food for Life Baking Co Inc	1.7	1.7	12	[1]
	Whole Grain Dinner Rolls	Udi's Gluten Free	1.6	1.6	12	[1]
	Toast		1.6	1.6	12	[10]

	Rolls, Whole Grain, Made with Tapioca Starch and Brown Rice Flour		1.6	1.6	12	[1]
	Rice Bread with Milk and Seeds	Rice Flour, Potato Starch, Corn Starch, Milk Powder, Seeds (Poppy, Amaranth, Flax, Sunflower, Pumpkin, Hazelnuts)	1.5	1.5	10	[16]
	Corn Dogs	Foster Poultry Farms	1.4	1.4	10	[1]
	Crispbread		1.4 (0.8–2.6) [1]	1.4 (0.8–2.6) [1]	11 (1–19) [1]	[10,11,15]
	Bread, Flax, Vegan	The Bakery	1.4	1.4	10	[18]
	Bread, With Added Grains, Commercial		1.3	1.3	10	[12]
	Bread, Homemade from Basic Ingredients		1.3	1.3	9	[12]
	Industrial Bread		1.3	1.3	9	[28]
	Bread, Cinnamon Raisin		1.2	1.2	9	[1]
	Rice Bread With Milk	Rice Flour, Potato Starch, Corn Starch, Milk Powder	1.2	1.2	9	[16]
	Buckwheat Bread	Buckwheat Flour, Potato Starch, Corn Starch	1.2	1.2	8	[16]
	Artisan Baker White Bread	Dr. Schar Usa, Inc.	1.2	1.2	8	[1]
	Bread	GF Wheat, Millet Flour; Corn Starch	1.1	1.1	8	[19]
	Bread, Cinnamon–Raisin	The Essential Baking Company, Inc.	1.1	1.1	8	[1]
	Sunny Seeded White, Bread	The Essential Baking Company, Inc.	1.1	1.1	8	[1]
	Soft & Hearty Whole Grain Bread	Pinnacle Foods Group Llc	1.1	1.1	8	[1]
	Original Cracklebred	Breton Enterprises Inc	1.1	1.1	8	[1]
	Bagels, Plain	Katz	1.0	1.0	7	[18]
	Bagels, Poppy Seed	Katz	1.0	1.0	7	[18]
	Multigrain Bread	Wal–Mart Stores, Inc.	1.0	1.0	7	[1]
	White Bread	The Kroger Co.	1.0	1.0	7	[1]
	7 Grain Bread	The Kroger Co.	1.0	1.0	7	[1]
	Bread, Commercial		1.0	1.0	7	[12]

	Millet Sandwich Bread	Brittany Records	1.0	1.0	7	[1]
	Bakehouse, Hamburger Buns	Harry's Farmers Market, Ltd.	1.0	1.0	7	[1]
	Ciabatta		1.0	1.0	7	[10]
	Raisin Brea		1.0	1.0	7	[10]
	Wholemeal Bread		1.0	1.0	7	[11]
	Oft & Chewy Bagels, Cinnamon Raisin	Pinnacle Foods Group Llc	0.9	0.9	7	[1]
	7 Grain Bread	United States Bakery	0.9	0.9	6	[1]
	Swiss Roll	Egg Without Shell, Sugar, Corn Flour, Potato Starch, Baking Powder, Mixed Berry Jam	0.9	0.9	6	[17]
	Bread	Semper	0.9	0.9	6	[9]
	GF Wheat–Free, Classic White Bread	Andrea's	0.9	0.9	6	[1]
	Rolls, White, Made with Brown Rice Flour, Tapioca Starch, And Sorghum Flour		0.9	0.9	6	[1]
	Bread with Raisins and Pecan	Food for Life Baking Co Inc	0.8	0.8	6	[1]
	Exotic Black Rice Bread	Food for Life Baking Co Inc	0.8	0.8	6	[1]
	Bread, Bhutanese Red Rice	Food for Life Baking Co Inc	0.8	0.8	6	[1]
	Soft & Hearty Whole Grain Bread	Udi's Gluten Free	0.8	0.8	6	[18]
	Classic French Dinner Rolls	Udi's Gluten Free	0.8	0.8	6	[1]
	Rolls, White, Made with Brown Rice Flour, Tapioca Starch, And Potato Starch		0.8	0.8	6	[1]
	Soft Dinner Roll	Rudi's Bakery	0.8	0.8	6	[1]
	Bread, White, Made with Potato Extract, Rice Starch, And Rice Flour		0.8	0.8	6	[1]

Bread, Whole Grain, Made with Tapioca Starch and Brown Rice Flour		0.8	0.8	6	[1]
Millet Sandwich Bread	Nation	0.8	0.8	6	[18]
Chestnut Bread		0.8	0.8	6	[15]
Raisin Bread		0.8	0.8	6	[15]
Scone		0.8	0.8	6	[10]
White Bread		0.7	0.7	5	[15]
Soft & Sweet Cinnamon & Raisin Bread	Pinnacle Foods Group Llc	0.7	0.7	5	[1]
Flatbread Pita	Entertainment Production House Inc.	0.7	0.7	5	[1]
Rice Bread	Rice Flour, Potato Starch, Corn Starch	0.7	0.7	5	[16]
Baquette		0.7	0.7	5	[10]
Shortbread		0.7	0.7	5	[10]
Bun		0.6	0.6	5	[10]
Multigrain Hot Dog Rolls	Purple Hands Llc	0.6	0.6	4	[1]
Bread,		0.6	0.6	4	[20]
Crispy & Delicious French Baguettes	Pinnacle Foods Group Llc	0.6	0.6	4	[1]
Bread, White, Made With Tapioca Starch And Brown Rice Flour		0.5	0.5	4	[1]
Bread, Multigrain Made with Real Honey	Rudi's Bakery	0.5	0.5	4	[18]
White Bread Carob Bean Gum		0.5	0.5	4	[15]
Brown Bread Carob Bean Gum		0.5	0.5	4	[15]
Sandwich Bread, Whit	Nation	0.5	0.5	4	[1]
White Sandwich Bread	Pinnacle Foods Group Llc	0.5	0.5	3	[1]

	Bread	Corn, GF Wheat	0.4	0.4	3	[5]
	Lye Pretzel		0.4	0.4	3	[10]
	Rustic Bread		0.4	0.4	3	[10]
	Soft & Hearty Bread Whole Grain Bread	Udi's Gluten Free	0.2	0.2	1	[1]
	Soft & Delicious White Sandwich	Udi's Gluten Free	0.2	0.2	1	[1]
	Brioche Bread		0.0	0.0	0	[11]
	Sliced White Bread (Sandwich)		0.0	0.0	0	[11]
	Bread Prepared with Olives		0.0	0.0	0	[11]
	White Bread (I, II)		0.0	0.0	0	[11]
	Bread Prepared with Oil		0.0	0.0	0	[11]
	Ciabatta Bread		0.0	0.0	0	[11]
	Piadina		0.0	0.0	0	[11]
	Melba Toast					
	No Data For 5 Products					
**IV**	Kellogg Special K Cereal	Kellogg Company Us, Fortified	18.4	5.5	39	[1]
	Breakfast Cereals, Nesquik	Nestlé Belgilux S.A.	11.0	3.3	24	[21]
	Oat Musli With Fruits	Oat (55%), Fruits (37%), Dates, Raisins, Sunflower Seeds	9.2	2.7	20	[5]
	Cerelac	Nestlé Belgilux S.A.	7.5	2.3	16	[21]
	Muesli	Muesli Fusion, Inc.	6.6	2.0	14	[1]
	Amaranth Flakes		6.4	1.9	14	[3]
	Amaranth And Quinoa Flakes		6.3	1.9	14	[3]
	Amaranth Flakes		6.1	1.8	13	[5]
	Cherry Walnut Granola Clusters	Pinnacle Foods Group Llc	6.0	1.8	13	[1]
	Wild Rice Flakes	From Hungary	5.8	1.7	12	[22]
	Instant Oatmeal	Glutenfreeda Foods, Inc.	5.6	1.7	12	[18]

Millet Musli		5.6	1.7	12	[15]
Freedom Muesli	Surf City Cereal	5.6	1.7	12	[1]
Corn Flakes		5.1	1.5	11	[28]
Granola	Georgia Peach Products, Inc.	5.1	1.5	11	[1]
Oat Flakes		4.9	1.5	10	[5]
Musli With Amaranth	Corn, Rice, Amaranth, Millet, Raisins, Raspberry	4.8	1.4	10	[5]
All–Purpose Flour	The King Arthur Flour Company, Inc.	4.8	1.4	10	[1]
Blueberry Cashew Granola Clusters	Pinnacle Foods Group Llc	4.8	1.4	10	[1]
Superfood Oatmeal	Nature's Path Organic	4.7	1.4	10	[1]
Granola	Garden Of Light Inc.	4.7	1.4	10	[1]
Oatmeal, Cinnamon Pumpkin Seed	Nature's Path Foods Inc.	4.7	1.4	10	[1]
Wild Rice Flakes	From Cambodia	4.7	1.4	10	[22]
Wild Rice Flakes	From Greece	4.7	1.4	10	[22]
Oats	Glutenfreeda Foods, Inc.	4.5	1.4	10	[1]
Oat Bran	Bob's Red Mill Natural Foods, Inc.	4.5	1.4	10	[1]
Homestyle Hot Oatmea	Nature's Path Foods Inc.	4.5	1.4	10	[1]
Hot Oatmeal, Brown Sugar Maple with Ancient Grains		4.5	1.4	10	[1]
Muesli, Commercial,		4.2	1.3	9	[12]
Instant Oatmeal, Natural	Glutenfreeda Foods, Inc.	4.2	1.3	9	[18]
Granola	Oat, Honey, Flax, Sesame, Pumpkin, Sunflower	3.9	1.2	8	[1]
Organic Superfood Oatmeal		3.8	1.1	8	[1]
Millet Flakes		3.8	1.1	8	[5]
Granola (including Original, Cranberry and Au Naural)	Pinnacle Foods Group Llc	3.6	1.1	8	[1]
Quick 1– Minute Oats	The Quaker Oats Company	3.6	1.1	8	[1]

4–Grain Flakes	Oat Flakes, Corn Flakes, Rice Flour, Miller Flour	3.5	1.1	8	[17]
Sorghum Flakes	Variety: Is8237 and Libery	3.5	1.0	7	[23]
Wild Rice Flakes	From Canada	3.4	1.0	7	[22]
Instant Oatmeal	The Quaker Oats Company	3.4	1.0	7	[1]
Oatmeal	Bob's Red Mill Natural Foods, Inc.	3.3	1.0	7	[1]
Cranberry Nut Crisp Granola	Delhaize America, Inc.	3.2	1.0	7	[1]
Instant Oatmeal	The Quaker Oats Company	3.1	0.9	7	[1]
Musli		3.0	0.9	6	[11]
GF Wheat Free, Instant Oatmeal, Strawberries	Glutenfreeda Foods, Inc.	2.9	0.9	6	[18]
Sorghum Flakes	Variety: Alpha	2.9	0.9	6	[23]
Oatmeal (including Strawberries & Brown Sugar W/ Flax Oatmeal, Blueberries & Cinnamon W/ Flax Oatmeal)	Glutenfreeda Foods, Inc.	2.8	0.8	6	[18]
Oats, Maple Raisin with Flax	Glutenfreeda Foods, Inc.	2.8	0.8	6	[18]
Granola (including Apple Almond Honey)	Glutenfreeda Foods, Inc.	2.6	0.8	6	[1]
Amaranth Musli With Fruits	Oat, Amaranth (20%), Fruits (20%), Hazelnut, Sesame, Buckwheat	2.5	0.8	5	[5]
Granola	Garden Of Light Inc.	2.4	0.7	5	[1]
Granola	Garden Of Light Inc.	2.4	0.7	5	[1]
Buckwheat Flakes		2.3	0.7	5	[5]
Rolled Oats	Glutenfreeda Foods, Inc.	2.2	0.7	5	[18]
Oatmeal	Topco Associates, Inc.	2.2	0.7	5	[1]
Bread Mix	Hodgson Mill	2.2	0.7	5	[1]
Steel Cut Oats	Glutenfreeda Foods, Inc.	2.1	0.6	5	[18]
Old Fashioned Oats Oatmeal	Topco Associates, Inc	2.1	0.6	4	[1]

	Mixed Berry Instant Oatmeal	Topco Associates, Inc.	1.9	0.6	4	[1]
	Granola (Nuts)		1.7	0.5	4	[10]
	Granola (Chocolate)		1.5	0.5	4	[10]
	Granola, Cranberry Pecan	Georgia Peach Products, Inc.	1.3	0.4	3	[1]
	Corn Flakes, Wheat Free Cereal	Nature's Path	1.2	0.4	3	[1]
	Cornflakes		1.2 (0.8–1.5) [1]	0.4 (0.2–0.5) [1]	3 (2–4) [1]	[3–7,10]
	Rice Flakes		0.4	0.1	0	[5]
	No Data For 1 Product					
**V**	Brown Rice & Chestnut Snack	Brown Rice, Chestnut Flour (60%)	9.5	4.7	34	[25]
	Amaranth Groats		8.9	4.5	32	[3]
	Tapioca		7.8	3.9	28	[3]
	Amaranth	Expanded Seeds	7.4	3.7	26	[3]
	Brown Rice & Chestnut Snack	Brown Rice, Chestnut Flour (40%)	6.9	3.4	24	[25]
	Amaranth Popping		5.7	2.9	20	[5]
	Brown Rice & Chestnut Snack	Brown Rice, Chestnut Flour (20%)	5.2	2.6	19	[25]
	Brown Rice Snack	Brown Rice	5.1	2.6	18	[25]
	Culinary Ancient Grains	Quinoa Corporation	5.0	2.5	18	[1]
	Millet Groats		4.8	2.4	17	[3]
	Ancient Quinoa Harvest Quinoa	Quinoa Corporation	4.8	2.4	17	[1]
	Whole Grain Steel Cut Oats	Bob's Red Mill Natural Foods, Inc.	4.6	2.3	16	[1]
	Steel Cut Oats	Oat	4.5	2.3	16	[1]
	Old Fashioned Oats	Country Choice Organic	4.5	2.3	16	[1]
	Sorghum		4.4	2.2	16	[3]
	Happy Quick Oats	Garden Of Light Inc.	4.8	2.4	17	[1]
	Quinoa Groats		3.7	1.9	13	[3]

	Buckwheat Groats		2.8	1.4	10	[3]
	Cous–Cous		2.0	1.0	7	[11]
	Rough Rice		1.5	0.8	5	[24]
	Brown Rice		1.3	0.7	5	[3]
	Cous–Cous		1.1 (0.4–0.2) [1]	0.6 (0.2–1.0) [1]	4 (2–7) [1]	[8,10,11]
	Rice Cakes with Quinoa	Rice, Quinoa (5%)	0.8	0.4	3	[5]
	Milled Rice		0.8	0.4	3	[24]
	Rice		0.8	0.4	3	[3]
	Corn Couscous		0.5	0.3	2	[28]
	No Data For 3 Products					
**VI**	Pasta	Millet–Pomace	42.9	21.5	153	[26]
	Pasta	Corn, Broad Bean (30%)	8.5	4.2	30	[29]
	Pasta With Teff	Corn, Teff (31%)	8.0	4.0	29	[5]
	Pasta	Extruded Rice (75%), Amaranth (25%)	7.6	3.8	27	[27]
	Pasta	Rice (75%), Amaranth (25%)	7.5	3.8	27	[27]
	Pasta	Extruded Rice (75%), Extruded Amaranth (25%)	7.5	3.8	27	[27]
	Rice Lasagna	The Hain Celestial Group, Inc.	6.3	3.2	23	[1]
	Pasta	The Hain Celestial Group, Inc.	6.3	3.2	23	[1]
	Pasta	Corn, Quinoa (20%)	5.9	2.9	21	[29]
	Pasta	Corn	3.9	2.0	14	[29]
	Vermicelli		3.8	1.9	14	[10]
	Tagliatelli		3.7	1.8	13	[10]
	Penne		3.2	1.6	11	[10]
	Garden Pagodas Organic Supergrain Pasta	Quinoa Corporation	3.2	1.6	11	[1]
	Soya Pasta		2.7	1.4	10	[3]
	Spaghetti		2.4	1.2	9	[10]
	Pasta	Brown Rice (87%), Corn (13%)	2.0	1.0	7	[13],

Wholemeal Pasta		2.0	1.0	7	[11]
Corn Pasta		2.0	1.0	7	[11]
Organic Elbows	Euro–Usa Trading Co., Inc.	1.9	0.9	7	[1]
Linguini	Euro–Usa Trading Co., Inc.	1.9	0.9	7	[1]
Fusilli		1.8	0.9	6	[10]
Pasta	Extruded Rice	1.7	0.9	6	[27]
Pasta	Rice	1.6	0.8	6	[27]
Brown Rice Pasta		1.5	0.7	5	[5]
Pasta	Brown Rice (60%), Corn (40%)	1.4	0.7	5	[13]
Lasagne Sheets		1.4	0.7	5	[10]
Pasta, Spaghetti,	Semper	1.3	0.7	5	[9]
Rice Pasta		1.0	0.5	4	[3]
Pasta Linguine		1.0	0.5	4	[28]
Egg Pasta		1.0	0.5	4	[11]
Pasta Made with Different Flours		1.0	0.5	4	[11]
Pasta For Broth		1.0	0.5	4	[11]
Pasta with Buckwheat Flour	Corn, Rice, Buckwheat (15%)	0.5	3		[5]
Pasta	Schar	0.9	0.5	3	[14]
Corn Pasta		0.9	0.5	3	[3]
Fresh Fusilli	Rp's Fresh Pasta	0.9	0.4	3	[1]
Pasta		0.9	0.4	3	[12]
Pasta, Made of Rice And Vegetables,	Orgran Tricolore	0.8	0.4	3	[9]
Fresh Linguini	Rp's Fresh Pasta	0.7	0.4	3	[1]
Organic Pipette – Fancy Elbows	Dakota Growers Pasta Company, Inc.	0.7	0.4	3	[1]
Spaghetti,	George Delallo Co., Inc.	0.6	0.3	2	[1]
Pasta	The Kroger Co.	0.6	0.3	2	[1]
Penne		0.6	0.3	2	[1]

Elbow Pasta	New World Pasta Company	0.6	0.3	2	[1]
Spaghetti No. 4, Pasta	George Delallo Co., Inc.	0.6	0.3	2	[1]
Elbows Corn & Rice Pasta Blend	Target Stores	0.6	0.3	2	[1]
Corn & Rice Pasta Blend	American Italian Pasta Company	0.6	0.3	2	[1]
Multigrain Spaghetti with Quinoa Pasta	Target Stores	0.6	0.3	2	[1]
Penne (Corn and Rice)		0.6	0.3	2	[1]
Pasta		0.5 (6–143) [1]	0.3 (6–143) [1]	1.7 (1–18) [1]	[1,15,28]
Pasta Fusilli		0.5	0.2	2	[28]
Pasta D'oro Linguine		0.5	0.2	2	[1]
Organic Rotini	Dakota Growers Pasta Company, Inc.	0.4	0.2	1	[1]
Organic Spaghetti	Dakota Growers Pasta Company, Inc.	0.4	0.2	1	[1]
Pasta	Corn, Rice	0.4	0.2	1	[5]
Rotini Pasta	New World Pasta Company	0.4	0.2	1	[1]
Pasta	New World Pasta Company	0.4	0.2	1	[1]
Pasta, Made of Corn and Rice	Barilla	0.3	0.2	1	[9]
Buckwheat Pasta		0.3	0.2	1	[3]
Rice Pasta		0.0	0.0	0	[11]
No Data For 6 Products					

[1] The average, minimum and maximum content/RNI of mineral in the same product from different references.

Table S3. Magnesium in gluten–free I – flours, II – mixes for cooking, III – bakery products, IV – cereals and flakes, V – groats and rice, VI – pasta.

	PRODUCT	PRODUCER/INGREDIENTS/OTHER INFORMATION	MG 100 G	MG PORTION	RNI PORTION	REFERENCE
I	Amaranth Flour Roasted		250	75	20	[3]
	Amaranth Flour		206 (84–269) [1]	62 (25–81) [1]	17 (7–22) [1]	[2–5]
	Quinoa		170(160–230) [1]	51 (48–69) [1]	14 (13–18) [1]	[6–8]

	Teff		165 (137–184) [1]	50 (41–55) [1]	14 (11–15) [1]	[3,5–7]
	Brown Rice Flour		128	38	10	[13]
	Buckwheat		128 (2–219) [1]	39 (1–66) [1]	10 (7–18) [1]	[3,5–7,30]
	Millet		122 (107–167) [1]	37 (32–49) [1]	10 (9–13) [1]	[3,5]
	Wholemeal Flour	Rice	112	34	9	[24]
	Chickpea		103 (20–166) [1]	31 (36–50) [1]	8 (10–13) [1]	[3,5]
	Oat		86 (39–133) [1]	26 (12–40) [1]	7 (3–11) [1]	[3,5–7]
	Sorghum Flour		85	25	7	[6,7]
	Chestnut		75 (74–76) [1]	23 (22–23) [1]	6 (6) [1]	[3,5]
	Flour	Jyttemel Original	68	20	5	[9]
	Acorn Flour		54	16	4	[5]
	Corn		33 (7–58) [1]	10 (2–17) [1]	3 (1–5) [1]	[5–7,13]
	Rice		31 (19–36) [1]	10 (6–11) [1]	3 (2–3) [1]	[3,5–7]
	Flour With 10% Of Oat Flour	Corn, Potato, Oat	23	7	2	[5]
	Flour, Mix	Rice, Soy, Corn, Tapioca	23	7	2	[12]
	Flour	Schär	16	5	1	[9]
	Flour, Coarse	Semper	14	4	1	[9]
	Rice Starch		6	2	0	[3]
	Corn Starch		3	1	0	[3]
	Brown Rice Flour		2	1	0	[30]
	No Data For 21 Products					
II	Multigrain Mix For Bread	Corn, Rice, Linseed Flour (6%), Sunflower Seeds (6%)	88	26	7	[5]
	Oat Mix For Bread	Oat (61%), Potato	72	22	6	[5]
	Oat Mix for Chocolate Muffins	Oat (20%), Potato, Rice	54	16	4	[5]
	Oat Cookies Mix	Oat (45%), Rice, Corn	53	16	4	[5]
	Baking Mix Brown	Glutafin	52	16	4	[14]
	Muffin Mix	Corn, Buckwheat	46	14	4	[5]
	Baking Mix White	Glutafin	12	4	1	[14]

	Flour Mix for Bread		11	3		1	[15]
	Baking Mix White	Schar	9	3		1	[14]
	Cake Base, Powder	Toro	9	3		1	[9]
	Cake Flour Mix		8	2		1	[15]
	Bread Mix	Corn, GF Wheat	7	2		1	[5]
	Cake Mix, Powder, Toro	Toro	7	2		1	[9]
	Flour Mix		4	1		0	[15]
	No Data For 66 Products						
**III**	Crisp Bread, Prepared from Powder	Toro	225		225	60	[9]
	Seed Crispbread	Buckwheat Flour, Sunflower Seed, Pumpkin Seed, Sesame Seed, Rapeseed Oil, Linseed, Salt	181		181	48	[17]
	Crispbread		153		153	41	[15]
	Bread, Homemade from Basic Ingredients		101		101	27	[12]
	Whole Grain Dinner Rolls	Udi's Gluten Free	96		96	26	[1]
	Rolls, Whole Grain, Made with Tapioca Starch and Brown Rice Flour		96		96	26	[1]
	Multigrain Bread	Corn, GF Wheat with Linseed, Sunflower, Soya, And Pumpkin Seeds	76		76	20	[5]
	Buckwheat Bread With Milk	Buckwheat Flour, Potato Starch, Corn Starch, Milk Powder	72		72	19	[16]
	Buckwheat Bread with Milk and Seeds	Buckhweta Flour, Potato Starch, Corn Starch, Milk Powder, Seeds (Poppy, Amaranth, Flax, Sunflower, Pumpkin, Hazelnuts)	71		71	19	[16]
	Bread With Grains	Corn, GF Wheat with Linseed, And Sunflower Seeds	69		69	18	[5]
	Bread With Oilseeds		64		64	17	[15]

Multigrain Bread	Corn, GF Wheat with Linseed (2.7%), Sunflower (2.7%), And Pumpkin Seeds (2.7%)	60	60	16	[5]
Millet Bread		56	56	15	[15]
Bread, With Added Grains, Commercial		52	52	14	[12]
Buckwheat Bread	Buckwheat Flour, Potato Starch, Corn Starch	49	49	13	[16]
Omega Flax Bread	Silver Hills	46	46	12	[1]
Classic French Dinner Rolls	Udi's Gluten Free	44	44	12	[1]
Rolls, White, Made with Brown Rice Flour, Tapioca Starch, And Potato Starch		44	44	12	[1]
Rice Bread with Milk and Seeds	Rice Flour, Potato Starch, Corn Starch, Milk Powder, Seeds (Poppy, Amaranth, Flax, Sunflower, Pumpkin, Hazelnuts)	43	43	11	[16]
Wheat–Free, Classic White Bread	Andrea's Gluten Free	42	42	11	[1]
Rolls, White, Made with Brown Rice Flour, Tapioca Starch, And Sorghum Flour		42	42	11	[1]
Bread Crumbs		41	41	11	[15]
Rice Bread With Milk	Rice Flour, Potato Starch, Corn Starch, Milk Powder	38	38	10	[16]
Brown Bread	Rice, Corn, Potato	35	35	9	[5]
Bread Brown	Glutafin	33	33	9	[14]
Bread, Whole Grain, Made with Tapioca Starch and Brown Rice Flour		31	31	8	[1]
Crisp Bread	Fette Croccanti	30	30	8	[14]

Bread, White, Made With	Tapioca Starch, Brown Rice Flour	25	25	7	[1]
Bread, Commercial		23	23	6	[12]
Bread	GF Wheat, Millet Flour; Corn Starch	18	18	5	[19]
Bread,		18	18	5	[20]
Bread	Semper	18	18	5	[9]
Bread	Corn, GF Wheat	15	15	4	[5]
Chestnut Bread		14	14	4	[15]
Original Sandwich Bread	Rudi's Bakery	13	13	3	[1]
Classic White Rolls	Dr. Schar Usa, Inc.	13	13	3	[1]
Bread, White, Made with Rice Flour, Corn Starch, and/Or Tapioca		12	12	3	[1]
Soft Dinner Roll	Rudi's Bakery	11	11	3	[1]
Bread, White, Made with Potato Extract, Rice Starch, And Rice Flour		11	11	3	[1]
Rice Bread	Rice Flour, Potato Starch, Corn Starch	11	11	3	[16]
Raisin Bread		11	11	3	[15]
White Bread		10	10	3	[15]
Rolls, White, Made with Rice Flour, Rice Starch, And Corn Starch		10	10	3	[1]
Bread White	Glutafin	10	10	3	[14]
Swiss Roll,	Egg Without Shell, Sugar, Corn Flour, Potato Starch, Baking Powder, Mixed Berry Jam	10	10	3	[17]
Brown Bread with Carob Gum		9	9	2	[15]
White Bread with Carob Gum		9	9	2	[15]

	Soft & Hearty Whole Grain Bread	Udi's Gluten Free	8	8	2	[1]
	Soft & Delicious White Sandwich Bread	Udi's Gluten Free	7	7	2	[1]
	No Data For 73 Products					
**IV**	Amaranth Flakes		301	90	24	[3]
	Wild Rice Flakes	From Hungary	300	90	24	[22]
	Amaranth And Quinoa Flakes		282	85	23	[3]
	Wild Rice Flakes	From Greece	249	75	20	[22]
	Amaranth Flakes		181	54	14	[5]
	Buckwheat Flakes		178	53	14	[5]
	Millet Flakes		132	40	11	[5]
	Oat Flakes		121	36	10	[5]
	Instant Oatmeal	The Quaker Oats Company	114	34	9	[1]
	Amaranth Musli with Fruits	Oat, Amaranth (20%), Fruits (20%), Hazelnut, Sesame, Buckwheat	114	34	9	[5]
	Oat Musli With Fruits	Oat (55%), Fruits (37%), Dates, Raisins, Sunflower Seeds	111	33	9	[5]
	Muesli, Commercial		110	33	9	[12]
	Kellogg Special K Cereal	Kellogg Company Us. Fortified with Fe	109	33	9	[1]
	Wild Rice Flakes	From Cambodia	101	30	8	[22]
	Quick 1– Minute Oats	The Quaker Oats Company	100	30	8	[1]
	Musli With Amaranth	Corn, Rice, Amaranth, Millet, Raisins, Raspberry	98	29	8	[5]
	4–Grain Flakes	Oat Flakes, Corn Flakes, Rice Flour, Miller Flour	98	29	8	[17]
	Instant Oatmeal	The Quaker Oats Company	93	28	7	[1]
	Wild Rice Flakes	From Canada	82	25	7	[22]
	Cranberry Nut Crisp Granola	Delhaize America, Inc.	71	21	6	[1]
	Rice Flakes		32	10	3	[5]
	Corn Flakes		6	2	0	[3]
	No Data For 51 Products					

**V**	Amaranth Groats		313	157	42	[3]
	Amaranth	Expanded Seeds	285	143	38	[3]
	Tapioca		239	120	32	[3]
	Amaranth Popping		223	112	30	[5]
	Buckwheat Groats		218	109	29	[3]
	Sorghum		162	81	22	[3]
	Rough Rice		143	72	19	[24]
	Quinoa Groats		131	66	17	[3]
	Rice Cakes With Quinoa	Rice, Quinoa (5%)	118	59	16	[5]
	Brown Rice		110	55	15	[3]
	Millet Groats		100	50	13	[3]
	Milled Rice		25	13	3	[24]
	Rice		13	7	2	[3]
	Brown Rice Snack	Brown Rice	12	6	2	[25]
	Brown Rice & Chestnut Snack	Brown Rice, Chestnut Flour (40%)	8	4	1	[25]
	Brown Rice & Chestnut Snack	Brown Rice, Chestnut Flour (20%)	8	4	1	[25]
	Brown Rice & Chestnut Snack	Brown Rice, Chestnut Flour (60%)	5	3	1	[25]
	No Data For 13 Products					
**VI**	Brown Rice Pasta		121	61	16	[5]
	Pasta, Spaghetti	Semper	120	60	16	[9]
	Pasta	Corn	119	60	16	[1]
	Buckwheat Pasta		111	56	15	[3]
	Pasta	Brown Rice (87%), Corn (13%)	107	54	14	[13]
	Pasta With Teff	Corn, Teff (31%)	86	43	11	[5]
	Pasta	Brown Rice (60%), Corn (40%)	78	39	10	[13]
	Pasta With Buckwheat Flour	Corn, Rice, Buckwheat (15%	72	36	10	[5]
	Soya Pasta		65	33	9	[3]

	Pasta, Made of Rice and Vegetables	Orgran Tricolore	51	26	7	[9]
	Pasta	Schar	31	16	4	[14]
	Pasta	Corn, Rice	23	12	3	[5]
	Pasta, Made of Corn and Rice	Barilla	17	9	2	[9]
	Rice Pasta		14	7	2	[3]
	Pasta		8 (2–13) [1]	4 (1–7) [1]	1 (0–2) [1]	[12,15]
	No Data For 54 Products					

[1] The average, minimum and maximum content/RNI of mineral in the same product from different references.

Table S4. Zinc in gluten–free I – flours, II – mixes for cooking, III – bakery products, IV – cereals and flakes, V – groats and rice, VI – pasta.

	PRODUCT	PRODUCER/INGREDIENTS/OTHER INFORMATION	MG 100 G	MG PORTION	RNI PORTION	REFERENCE
I	Amaranth		2.9 (1.8–4.2) [1]	0.9 (0.5–1.3) [1]	9 (5–13) [1]	[2,4,5]
	Chickpea Flour		2.9	0.9	9	[5]
	Teff		3.6 (2.5–4.2) [1]	1.0 (0.7–1.2) [1]	10 (7–12) [1]	[5–7]
	Quinoa		2.9 (2.4–3.3) [1]	0.9 (0.7–1.0) [1]	9 (7–10) [1]	[6–8]
	Millet Flour		2.2	0.7	7	[5]
	Buckwheat		2.1 (1.2–3.1) [1]	0.6 (0.3–0.9) [1]	6 (3–9) [1]	[5–7]
	Oat		2.1 (1.1–3.0) [1]	0.6 (0.3–1.9) [1]	3 (3–9) [1]	[5–7]
	Brown Rice Flour		1.8	0.5	5	[13]
	Flour	Jyttemel Original	1.5	0.5	5	[9]
	Rice		1.5 (1.1–1.8) [1]	0.4 (0.3–0.5) [1]	4 (3–5) [1]	[5–7,31]
	Chestnut Flour		1.1	0.3	3	[5]
	Sorghum Flour		1.0	0.3	3	[6,7]
	Flour		1.0	0.3	3	[10]
	Acorn Flour		0.8	0.2	2	[5]
	Corn		0.7 (0.2–1.1) [1]	0.2 (0.1–0.3) [1]	2 (1–3) [1]	[5–7,13]

	GF Flour with Oat Flour	Corn, Potato, Oat (10%)	0.6	0.2	2	[5]
	Flour	Schär	0.4	0.1	1	[9]
	Flour, Coarse	Semper	0.4	0.1	1	[9]
	Flour, Mix	Rice, Soy, Corn, Tapioca	0.3	0.1	1	[12]
	Flour		0.1	0.0	0	[28]
	Mixed Flours		0.0	0.0	0	[11]
	No Data For 45 Products					
**II**	Oat Mix For Bread	Oat (61%), Potato	1.9	0.6	6	[5]
	Breadcrumbs		1.9	0.6	6	[10]
	Bake mix (Pizza)		1.6	0.5	5	[10]
	Oat Cookies Mix	Oat (45%), Rice, Corn	1.3	0.4	4	[5]
	Oat Mix for Chocolate Muffins	Oat (20%), Potato, Rice	1.1	0.3	3	[5]
	Bake mix white (cake)		1.1	0.3	3	[10]
	Multigrain Mix For Bread	Corn, Rice, Linseed Flour (6%), Sunflower Seeds (6%)	1.0	0.3	3	[5]
	Flour Mix For Bread		1.0	0.3	3	[15]
	Cake Flour Mix		0.9	0.3	3	[15]
	Cake Base,	Toro	0.8	0.2	2	[9]
	Bake mix brown (cake)		0.8	0.2	2	[10]
	Muffin Mix	Corn, Buckwheat	0.5	0.1	1	[5]
	Flour For Cakes		0.0	0.0	0	[28]
	Baking Mix White	Schar	0.4	0.1	1	[14]
	Cake Mix	Toro	0.2	0.1	1	[9]
	Bread Mix	Corn, GF Wheat	0.2	0.0	0	[5]
	Baking Mix Brown	Glutafin	0.1	0.0	0	[14]
	Flour Mix		0.0	0.0	0	[15]
	Breadcrumbs		0.0	0.0	0	[11]
	Flour for Cake		0.0	0.0	0	[11]
	Flour for Bread		0.0	0.0	0	[11]

	No Data For 115 Products					
**III**	Shortbread		8.2	8.2	82	[28]
	Crisp Bread, Prepared from Powder	Toro	4.7	4.7	47	[9]
	Seed Crispbread	Buckwheat Flour, Sunflower Seed, Pumpkin Seed, Sesame Seed, Rapeseed Linseed	3.2	3.2	32	[17]
	Bread Crumbs		2.2	2.2	22	[15]
	Buckwheat Bread With Milk	Buckwheat Flour, Potato Starch, Corn Starch, Milk Powder	2.0	2.0	20	[16]
	Crisp Bread		2.9 (1.8–4.2) [1]	0.9 (0.5–1.3) [1]	9 (5–13) [1]	[2,4,5]
	Wholegrain Bread		1.7	1.7	12	[10]
	Millet Bread		1.6	1.6	16	[15]
	Bread, Homemade from Basic Ingredients		1.6	1.6	16	[12]
	Whole Grain Dinner Rolls	Udi’s Gluten Free's	1.4	1.4	3	[1]
	Rolls, Whole Grain	Tapioca Starch, Brown Rice Flour	1.4	1.4	3	[1]
	Bread with oilseeds		1.3	1.3	13	[15]
	Toast		1.2	1.2	12	[10]
	Rice Bread With Milk	Rice Flour, Potato Starch, Corn Starch, Milk Powder	1.1	1.1	11	[16]
	Buckwheat Bread	Buckwheat Flour, Potato Starch, Corn Starch	1.1	1.1	11	[16]
	Buckwheat Bread with Milk And Seeds	Buckwheat Flour, Potato Starch, Corn Starch, Milk Powder, Seeds (Poppy, Amaranth, Flax, Sunflower, Pumpkin, Hazelnuts)	1.0	1.0	10	[16]
	Multigrain Bread	Corn, GF Wheat with Linseed, Sunflower, Soya, Pumpkin Seeds	1.0	1.0	10	[5]
	Ciabatta		0.9	0.9	9	[10]
	Multigrain Bread	Corn, GF Wheat with Linseed (2.7%), Sunflower (2.7%), Pumpkin Seeds (2.7%)	0.9	0.9	9	[5]
	Bread, With Added Grains, Commercial		0.9	0.9	9	[12]
	Omega Flax Bread	Silver Hills	0.9	0.9	9	[1]
	Brown Bread	Rice, Corn, Potato	0.8	0.8	8	[5]
	Rice Bread With Milk And Seeds	Rice Flour, Potato Starch, Corn Starch, Milk Powder, Seeds (Poppy, Amaranth, Flax, Sunflower, Pumpkin, Hazelnuts)	0.8	0.8	8	[16]

Bread	Semper	0.8	0.8	8	[9]
Bread With Grains	Corn, GF Wheat with Linseed, Sunflower Seeds	0.8	0.8	8	[5]
Wheat–Free, Classic White Bread	Andrea's Gluten Free	0.8	0.8	8	[1]
Rolls, White	Brown Rice Flour, Tapioca Starch, Sorghum Flour	0.8	8	8	[1]
Rice Bread	Rice Flour, Potato Starch, Corn Starch	0.7	0.7	7	[16]
Classic French Dinner Rolls	Udi’s Gluten Free's Gluten Free	0.7	0.7	7	[1]
Rolls, White	Brown Rice Flour, Tapioca Starch, Potato Starch	0.7	0.7	7	[1]
Swiss Roll,	Corn Flour, Potato Starch	0.7	0.7	7	[17]
White Bread with Carob Gum		0.7	0.7	7	[15]
Brown Bread with Carob Gum		0.7	0.7	7	[15]
Raisin Bread		0.7	0.7	7	[10]
Short Bread		0.6	0.6	6	[10]
Lye Pretzel		0.6	0.6	6	[10]
Chestnut Bread		0.6	0.6	6	[15]
Crisp Bread	Fette Croccanti	0.6	0.6	6	[14]
Bread, Grain	Tapioca Starch, Brown Rice Flour	0.6	0.6	6	[1]
Original Sandwich Bread	RUdi’s Gluten Free's Bakery	0.6	0.6	6	[1]
Classic White Rolls	Dr. Schar Usa, Inc.	0.6	0.6	6	[1]
Bread, Commercial		0.5	0.5	5	[12]
Bread, White,	Tapioca Starch, Brown Rice Flour	0.5	0.5	5	[1]
Local Bread		0.5	0.5	5	[28]
Bread, White	Rice Flour, Corn Starch, Tapioca	0.5	0.5	5	[1]
Scone		0.5	0.5	5	[10]
Rustic Bread		0.5	0.5	5	[10]
Baquette		0.4	0.4	4	[10]

	Bun		0.4	0.4	4	[10]
	Rolls,	Rice Flour, Corn Starch, Tapioca	0.4	0.4	4	[1]
	Bread		0.4	0.4	4	[20]
	Bread	GF Wheat, Millet Flour; Corn Starch	0.4	0.4	4	[19]
	Local Bread B.		0.3	0.3	3	[28]
	Bread	Corn, GF Wheat	0.3	0.3	3	[5]
	Soft Dinner Roll	RUdi’s Gluten Free's Bakery	0.3	0.3	3	[1]
	Bread, White	Potato Extract, Rice Starch, Rice Flour	0.3	0.3	3	[1]
	Industrial Bread		0.2	0.2	2	[28]
			0.2	0.2	2	[10]
	Soft & Delicious White Sandwich Bread	Udi’s Gluten Free's	0.1	0.1	1	[1]
	Soft & Hearty Whole Grain Bread	Udi’s Gluten Free's	0.1	0.1	1	[1]
	Bread Brown	Glutafin	0.1	0.1	1	[14]
	Raisin Bread		0.1	0.1	1	[15]
	Brioche Bread		0.0	0.0	0	[11]
	Sliced Bread (Sandwich)		0.0	0.0	0	[11]
	Bread Prepared With Olives		0.0	0.0	0	[11]
	White Bread (I)		0.0	0.0	0	[11]
	Wholemeal Bread		0.0	0.0	0	[11]
	Bread Prepared with Oil		0.0	0.0	0	[11]
	Ciabattta Bread		0.0	0.0	0	[11]
	Piadina		0.0	0.0	0	[11]
	White Bread (II)		0.0	0.0	0	[11]
	Melba Toast		0.0	0.0	0	[11]
	No Data For 47 Products					
**IV**	Blueberry Cashew Granola Clusters	Udi’s Gluten Free's Gluten Free	12.5	3.8	38	[1]
	Wild Rice Flakes	From Hungary	4.5	1.4	3	[22]

	Oat Flakes		3.1	0.9	9	[5]
	Wild Rice Flakes	From Cambodia	3.0	0.9	9	[22]
	Wild Rice Flakes	From Greece	2.7	0.8	8	[22]
	4–Grain Flakes	Oat Flakes, Corn Flakes, Rice Flour, Miller Flour	2.6	0.8	8	[17]
	Millet Flakes		2.5	0.8	8	[5]
	Cerelac	Nestlé Belgilux S.A., Fortified	2.5	0.8	8	[21]
	Amaranth Flakes		2.4	0.7	7	[5]
	Buckwheat Flakes		2.4	0.7	7	[5]
	Oat Musli With Fruits	Oat (55%), Fruits (37%), Dates, Raisins, Sunflower Seeds	2.3	0.7	7	[5]
	Sorghum Flakes	Variety: Liberty	2.2	0.6	6	[23]
	Wild Rice Flakes	From Canada	2.0	0.6	6	[22]
	Muesli, Commercial		1.8	0.5	5	[12]
	Amaranth Musli With Fruits	Oat, Amaranth (20%), Fruits (20%), Hazelnut, Sesame, Buckwheat	1.7	0.5	5	[5]
	Sorghum Flakes	Variety: IS8237C	1.6	0.5	5	[23]
	Granola (nuts)		1.6	0.5	5	[10]
	Sorghum Flakes	Variety: Alpha	1.5	0.4	4	[23]
	Musli With Amaranth	Corn, Rice, Amaranth, Millet, Raisins, Raspberry	1.5	0.4	4	[5]
	Kellogg Special K	Kellogg Company Us, Fortified with Fe	1.4	0.4	4	[1]
	Rice Flakes		1.4	0.4	4	[5]
	Millet Musli		1.4	0.4	4	[15]
	Corn Flakes		0.9 (0.3–1.5) [1]	0.3 (0.1–0.4) [1]	2 (1–2) [1]	[10,28]
	No Data For 88 Products					
**V**	Amaranth Popping		3.1	1.5	15	[5]
	Brown Rice		2.3	1.1	11	[31]
	Rice Cakes with Quinoa	Rice, Quinoa (5%)	1.3	0.7	7	[5]
	White Rice		1.3	0.7	7	[31]
	Enriched White Rice		1.2	0.6	6	[31]
	Granola (Chocolate)		0.5	0.2	2	[10]

	Cous–cous		0.5 (0.2–1.0) [1]	0.3 (0.1–0.5) [1]	2 (1–4) [1]	[10,11,28]
	Corn Couscous		0.2	0.1	1	[28]
	Musli		0.0	0.0	0	[11]
	No Data For 21 Products					
**VI**	Pasta	Millet–Pomace	15.8	7.9	79	[26]
	Pasta	Extruded Rice (75%), Amaranth (25%)	7.3	3.7	37	[27]
	Pasta	Extruded Rice (75%), Extruded Amaranth (25%)	7.2	3.6	36	[27]
	Pasta	Rice (75%), Amaranth (25%)	7.1	3.6	36	[27]
	Vermicelli		3.1	1.5	15	[10]
	Tagliatelli		3.0	1.5	15	[10]
	Wholemeal Pasta		3.0	1.5	15	[11]
	Spaghetti		2.5	1.3	13	[10]
	Fusilli		1.9	1.0	10	[10]
	Pasta, Spaghetti	Semper	1.9	1.0	10	[9]
	Pasta	Corn	1.8	0.9	9	[1]
	Pasta	Corn, Broad Bean (30%)	1.7	0.9	9	[29]
	Brown Rice Pasta		1.7	0.8	8	[5]
	Pasta	Corn, Quinoa (20%)	1.5	0.8	8	[29]
	Pasta, Made of Rice and Vegetables	Orgran Tricolore	1.4	0.7	7	[9]
	Penne		1.4	0.7	7	[10]
	Lasagne Sheets		1.3	0.7	7	[10]
	Pasta with Teff	Corn, Teff (31%)	1.3	0.7	7	[5]
	Pasta with Buckwheat Flour	Corn, Rice, Buckwheat (15%	1.3	0.6	6	[5]
	Pasta	Brown Rice (87%), Corn (13%)	1.2	0.6	6	[13]
	Egg Pasta		1.0	0.5	5	[11]
	Pasta made with different Flours		1.0	0.5	5	[11]
	Corn Pasta		1.0	0.5	5	[11]

Rice Pasta		1.0	0.5	5	[11]
Pasta For Broth		1.0	0.5	5	[11]
Pasta	Brown Rice (60%), Corn (40%)	0.7	0.4	4	[13]
Pasta	Rice	0.7	0.4	4	[27]
Pasta	Extruded Rice	0.7	0.4	4	[27]
Pasta	Corn	0.5	0.3	3	[29]
Pasta	Schar	0.5	0.3	3	[14]
Pasta, Made of Corn and Rice	Barilla	0.4	0.2	2	[9]
Pasta	Corn, Rice	0.4	0.2	2	[5]
Pasta		0.4 (0.1–0.6) [1]	0.2 (0.1–0.3) [1]	2 (0–3) [1]	[15,28]
Pasta, Plain		0.3	0.2	1	[12]
Pasta Fusilli		0.3	0.2	1	[28]
Pasta Linguine		0.3	0.2	1	[28]
No data for 56 products					

[1] The average, minimum and maximum content/RNI of mineral in the same product from different references.

Reference

1. USDA Food Composition Database. Release April 2018. Available online: https://ndb.nal.usda.gov/ndb/search/list (accessed between 26 May and 10 October 2018).
2. Kumar, K.V.P.; Dharmaraj, U.; Sakhare, S.D.; Inamdar, A.A. Preparation of protein and mineral rich fraction from grain amaranth and evaluation of its functional characteristics. *J. Cereal Sci.* **2016**, *69*, 358–362, doi:10.1016/j.jcs.2016.05.002.
3. Kunachowicz, H. (ed.). 2015. *Gluten-Free Diet – What to Choose*? 2nd ed.; Wydawnictwo Lekarskie PZWL: Warsaw, Poland, 2015; pp. 99–170; ISBN 978-83-200-4965-7.
4. Sanz-Penella, J.M.; Wronkowska, M.; Soral-Smietana, M.; Haros, M. Effect of whole amaranth flour on bread properties and nutritive value. *LWT-Food Sci. Technol.* **2013**, *50*, 679–685, doi:10.1016/j.lwt.2012.07.031.
5. Rybicka, I.; Gliszczyńska-Świgło, A. Minerals in grain gluten-free products. The content of calcium, potassium, magnesium, sodium, copper, iron, manganese, and zinc. *J. Food Compost. Anal.* **2017**, *59*, 61–67, doi:10.1016/j.jfca.2017.02.006.
6. Hager, A.S.; Czerny, M.; Bez, J.; Zannini, E.; Arendt, E.K. Starch properties, in vitro digestibility and sensory evaluation of fresh egg pasta produced from oat, teff and wheat flour. *J. Cereal Sci.* **2013**, *58*, 156–163, doi:10.1016/j.jcs.2013.03.004.
7. Hager, A.S.; Wolter, A.; Jacob, F.; Zannini, E.; Arendt, E.K. Nutritional properties and ultra-structure of commercial gluten free flours from different botanical sources compared to wheat flours. *J. Cereal Sci.* **2012**, *56*, 239–247, doi:10.1016/j.jcs.2012.06.005.
8. Jan, R.; Saxena, D.C.; Singh, S. Comparative study of raw and germinated Chenopodium (Chenopodiumalbum) flour on the basis of thermal, rheological, minerals, fatty acid profile and phytocomponents. *Food Chem.* **2018**, *269*, 173–180, doi:10.1016/j.foodchem.2018.07.003.

9. Norwegian Food Composition Table Matvaretabellen. 2018. Available online: http://www.matvaretabellen.no/ (accessed between 26 May and 10 October 2018).
10. Missbach, B.; Schwingshackl, L.; Billmann, A.; Mystek, A.; Hickelsberger, M.; Bauer, G.; Konig, J. Gluten-free food database: The nutritional quality and cost of packaged gluten-free foods. *PeerJ* **2017**, 1–18, doi:10.7717/peerj.1337.
11. Mazzeo, T.; Cauzzi, S.; Brighenti, F.; Pellegrini, N. The development of a composition database of gluten-free products. *Public Health Nutr.* **203**, *18*, 1353–1357, doi:10.1017/S136898003001682.
12. Nutrient Reference Values for Australia and New Zealand. 2018. Available online: https://www.nrv.gov.au/nutrients-energy-calculation/nutrients-energy-calc-result-153302860 (accessed between 26 May and 10 October 2018).
13. Silva, E.M.M.; Ascheri, J.L.R.; Ascheri, D.P.R. Quality assessment of gluten-free pasta prepared with a brown rice and corn meal blend via thermoplastic extrusion. *LWT-Food Sci. Technol.* **2016**, *68*, 698–706, doi:10.1016/j.lwt.2015.12.067.
14. The Dutch Food Composition Database (NEVO) online version 2016/5.0. 2016. Available online: https://nevo-online.rivm.nl/ (accessed between 26 May and 10 October 2018).
15. Bundeslebensmittelschlüssel BLS, version 3.02. 2018. Available online: https://www.blsdb.de/ (accessed between 26 May and 10 October 2018).
16. Regula, J.; Cerba1, A.; Suliburska, J.; Tinkov, A. In Vitro bioavailability of calcium, magnesium, iron, zinc, and copper from gluten-free breads supplemented with natural additives. *Biol. Trace Elem. Res.* **2018**, *182*, 30–36, doi:10.1007/s12011-017-1065-4.
17. Fineli, version 29.06.2018. 2018. Available online: https://fineli.fi/fineli/en/index? (accessed between 26 May and 10 October 2018).
18. Nutritionix. 2018. Available online: https://www.nutritionix.com/ (accessed between 26 May and 10 October 2018).
19. Rizzello, C.G.; Montemurro, M.; Gobbetti, M. Characterization of the Bread Made with Durum Wheat Semolina Rendered Gluten Free by Sourdough Biotechnology in Comparison with Commercial Gluten-Free Products. *J. Food Sci.* **2016**, *81*, H2263–H2272, doi:10.1111/1750-3841.13410.
20. NutriData Estonian Food Composition Database, version 8. 2018. Available online http://tka.nutridata.ee/index.action?request_locale=en (accessed between 26 May and 10 October 2018).
21. Internubel 2018. Available online: http://www.internubel.be (accessed between 26 May and 10 October 2018).
22. Sumczynski, D.; Koubová, E.; Šenkárová, L.; Orsavová, J. Rice flakes produced from commercial wild rice: Chemical compositions, vitamin B compounds, mineral and trace element contents and their dietary intake evaluation. *Food Chem.* **2018**, *264*, 386–392, doi:10.1016/j.foodchem.2018.05.061.
23. Wu, G.; Ashton, J.; Simic, A.; Fang, Z.; Johnson, S.K. Mineral availability is modified by tannin and phytate content in sorghum flaked breakfast cereals. *Food Res. Int.* **2018**, *103*, 509–53, doi:10.1016/j.foodres.2017.09.050.
24. Rosell, C.M.; Marco, C. Rice. In *Gluten-Free Cereal Products and Beverages*; Arendt, E.K., Dal Bello, F. Academic Press Elsevier Inc.: Burlington, VT, USA, 2008; pp. 81–100. ISBN 978-0-12-373739-7.
25. Mir, S.A.; Bosco, S.J.D.; Shah, M.A. Technological and nutritional properties of gluten-free snacks based on brown rice and chestnut flour. *J. Saudi Soc. Agric. Sci.* **2017**, doi:10.1016/j.jssas.2017.02.002.
26. Gull, A.; Prasad, K.; Kumar, P. Nutritional, antioxidant, microstructural and pasting properties of functional pasta. *J. Saudi Soc. Agric. Sci.* **2018**, *17*, 37–153, doi:10.1016/j.jssas.2016.03.002.
27. Cabrera-Chávez, F.; Calderón de la Barca, A.M.; Islas-Rubio, A.R.; Marti, A.; Marengo, M.; Pagani, M.; Bonomi, F.; Iametti, S. Molecular rearrangements in extrusion processes for the production of amaranth-enriched, gluten-free rice pasta. *LWT-Food Sci. Technol.* **2012**, *47*, 421–426, doi:10.1016/j.lwt.2012.01.040.
28. Orecchio, S.; Amorello, S.; Raso, M.; Barreca, S.; Lino, C.; Di Gaudio, F. Determination of trace elements in gluten-free food for celiac people by ICP-MS. *Microchem. J.* **203**, *116*, 163–172, doi:10.1016/j.microc.203.04.011.

29. Giménez, M.A.; Drago, S.R.; Bassett, M.N.; Lobo, M.O.; Sammán, N.C. Nutritional improvement of corn pasta-like product with broad bean (Vicia faba) and quinoa (Chenopodium quinoa). *Food Chem.* **2016**, *199*, 150–156, doi:10.1016/j.foodchem.2015.11.065.
30. Markiewicz-Keszycka, M.; Casado-Gavaldaa, M.P.; Cama-Moncunilla, X., Cama-Moncunilla, R.; Dixita, Y.; Cullena, P.J.; Sullivana, C. Laser-induced breakdown spectroscopy (LIBS) for rapid analysis of ash, potassium and magnesium in gluten free flours. *Food Chem.* **2018**, *244*, 324–330, doi: 10.1016/j.foodchem.2017.10.063.
31. Punshon, T.; Jackson, B.P. Essential micronutrient and toxic trace element concentrations in gluten containing and gluten-free foods. *Food Chem.* 2018, *252*, 258–264, doi:10.1016/j.foodchem.2018.01.120.

© 2018 by the authors. Submitted for possible open access publication under the terms and conditions of the Creative Commons Attribution (CC BY) license (http://creativecommons.org/licenses/by/4.0/).
